# Local adaptation of *Gymnocypris przewalskii* (Cyprinidae) on the Tibetan Plateau

**DOI:** 10.1038/srep09780

**Published:** 2015-05-06

**Authors:** Renyi Zhang, Arne Ludwig, Cunfang Zhang, Chao Tong, Guogang Li, Yongtao Tang, Zuogang Peng, Kai Zhao

**Affiliations:** 1 Key Laboratory of Adaptation and Evolution of Plateau Biota, Northwest Institute of Plateau Biology, Chinese Academy of Sciences, Xining 810001, China; 2 Department of Evolutionary Genetics, Leibniz Institute for Zoo and Wildlife Research, Berlin 10324, Germany; 3 Key Laboratory of Freshwater Fish Reproduction and Development (Ministry of Education), Southwest University School of Life Sciences, Chongqing 400715, China; 4 University of Chinese Academy of Sciences, Beijing 100049, China

## Abstract

Divergent selection among environments affects species distributions and can lead to speciation. In this article, we investigated the transcriptomes of two ecotypes of scaleless carp (*Gymnocypris przewalskii przewalskii* and *G. p. ganzihonensis*) from the Tibetan Plateau. We used a transcriptome sequencing approach to screen approximately 250,000 expressed sequence tags (ESTs) from the gill and kidney tissues of twelve individuals from the Ganzi River and Lake Qinghai to understand how this freshwater fish has adapted to an ecological niche shift from saline to freshwater. We identified 9,429 loci in the gill transcriptome and 12,034 loci in the kidney transcriptome with significant differences in their expression, of which 242 protein-coding genes exhibited strong positive selection (*K*_a_/*K*_s_ > 1). Many of the genes are involved in ion channel functions (e.g., Ca^2+^-binding proteins), immune responses (e.g., nephrosin) or cellular water absorption functions (e.g., aquaporins). These results have potentially broad importance in understanding shifts from saline to freshwater habitats. Furthermore, this study provides the first transcriptome of *G. przewalskii*, which will facilitate future ecological genomics studies and aid in the identification of genes underlying adaptation and incipient ecological speciation.

The causes of speciation have long been one of the central questions in evolutionary biology^1^,^2^,^3^,^4^,^5^,^6^. When one population colonizes a new habitat or niche, they often diverge phenotypically and genetically to increase the fitness of the population^7^. Adaptation to different environments is underpinned by both regulatory variation and structural protein variation^8^,^9^. Therefore, linking fitness to genotypic variation is key to understanding local adaptation and ecological speciation^10^,^11^. However, the molecular mechanisms underlying adaptation in gene expression and coding sequence divergence to various environmental conditions in natural populations remain relatively poorly understood.

In the past, elucidating the genomic basis of adaptation and speciation has been a challenge, especially in natural systems, because of the lack of genomic resources for non-model organisms. Next generation sequencing (NGS) techniques provide new opportunities for studying the genomics of non-model organisms. In particular, transcriptome sequencing promises to reveal numerous aspects of ecological speciation^12^,^13^, such as discovering genes previously unaffiliated with ecological environments and examining the relative contributions of gene expression and coding sequence divergence to phenotypic changes apparent during ecological speciation.

Many of the salt lakes in China are located in the Tibetan Plateau, of which Lake Qinghai is the most prominent representative. Lake Qinghai is the biggest inland salt lake in China, characterized by high salt and alkali content (salinity 14 parts per thousand by weight; pH 9.3). Historically, Lake Qinghai was a freshwater lake connected to the Yellow River. Geologic data indicates that approximately 1.2 Ma BP the Yellow River emerged at the edge of the plateau, and approximately 0.15 Ma BP the “Gonghe Movement” of the Tibetan Plateau led to the separation of Lake Qinghai from the upper reaches of the Yellow River, after which Lake Qinghai became an occlusion lake^14^,^15^. Originally the fauna of both Yellow River and Lake Qinghai was similar, including many fish species (e.g., *Gymnocypris eckloni*, *Schizopygopsis pylzovi*, *Chuanchia labiosa*, *Platypharodon extremus*, *Gymnodiptychus pachycheilus*, *Triplophysa stoliczkai*, *T. dorsonotatus*, *T. scleroptera* and *T. siluroides*). As the climate turned colder and drier at the dawn of the Holocene (10,500 to 7,500 yr BP), the salinity and alkalinity of Lake Qinghai increased, and few fish species survived (*G. przewalskii*, *N. T. stoliczkai*, *T. dorsonotatus*, *T. scleroptera* and *T. alticeps*)^16^,^17^,^18^. Currently, the dominant species is the endemic, scaleless carp, *Gymnocypris przewalskii* (Kessler) (Cyprinidae: Schizothoracinae). The scaleless carp is a cold-water-adapted freshwater fish, narrowly distributed in Lake Qinghai Basin in China^19^. A subspecies or ecotype (*G. p. ganzihonensis*) of the scaleless carp has been identified in the Ganzi River (Fig. 1) based on the shape and number of gill rakers^16^. While historical sources indicate that the Ganzi River once flowed into Lake Qinghai, an additional survey, conducted in 1964, revealed that the lake lacked a connection to the Ganzi River, likely reflecting the shrinking of the lake shoreline and low flows in the upper reaches^16^. The upper reaches have evolved into a subterranean river where the water peters out and the scaleless carp colonized this small freshwater habitat^16^. Therefore, *G. przewalskii* is an excellent system to study adaptational differences between saline and freshwater habitats, especially when phenotypic divergence and speciation are the results of divergent natural selection.

In this study, we sequenced the transcriptomes of the two ecotypes of *G. przewalskii* inhabiting the freshwater Ganzi River and the saltwater Lake Qinghai to study the changes in their transcriptomes that may be adaptations to the different environments in which they live. These fish ecotypes are a unique opportunity to study the process of divergence and speciation in the harsh climate of the Tibetan Plateau.

## Results

### Sequencing and assembly

We constructed four cDNA libraries derived from gill and kidney tissues from both salt and freshwater ecotypes of *G. przewalskii*. Each sample resulted in a library containing an average of 80 million reads equal to 6 Gb and a total data of 24 Gb were generated. Two different strategies (separate or pooled assembly) were used to guarantee appropriate sequencing depth data for each assembly. The pooled assembly was used for the final analyses, and the average transcript length was 952 bp, with an N50 read length of 1,836 bp. The characteristics of the assembly are provided in Table S1.

### Functional annotation

To annotate the *G. przewalskii* transcriptome, all unigene sets were annotated based on similarity to sequences in several public databases. A total of 158,087 unigenes were queried against the Nr protein database, Swiss-Prot, KEGG, COG and nt database using BLASTX and BLASTN with an E-value cut-off of < 10^−5^. A search of the Nr protein database yielded 77,313 unigenes (48.9%; Table S2). The E-value distribution of the top hits in the Nr database ranged from 0 to 1.0E^−5^ (Fig. 2a). The similarity distribution of the top BLAST hits for each sequence ranged from 17–100% (Fig. 2b). The top BLAST hits matched annotated unigenes from *Danio rerio*, *Oreochromis niloticus*, *Tetraodon nigroviridis*, *Salmo salar* and *Xenopus tropicalis* (Fig. 2c). The genome annotations for these species are comprehensive and largely accepted, suggesting that the *G. przewalskii* sequences were correctly assembled and annotated.

Gene Ontology (GO) assignments were used to classify the functions of the predicted *G. przewalskii* genes. Based on sequence homology, 51,671 sequences were categorized into 52 functional groups in three functional divisions (Fig. 3). Many of the unigenes were classified into “cellular process”, “cell” and “cell part”, whereas only a few genes belonged to “carbohydrate utilization”, “nitrogen utilization”, “protein tag”, “morphogen activity” and “metallochaperone activity”. Intriguingly, 157 unigenes were classified into “channel regulator activity”, while only 44 unigenes were classified into “translation regulator activity” categories.

To further evaluate the integrity of the transcriptome library and determine the effectiveness of the annotation process, unigene sequences were subjected to COG classification. Among 77,313 Nr hits, 25,272 sequences were assigned a COG classification (Fig. 4). Among the 25 COG categories, the cluster for “general function prediction only” was the largest group (10,768 unigenes), followed by “replication, recombination and repair” (6,313 unigenes), “transcription” (5,086 unigenes), “signal transduction mechanisms” (3,962 unigenes), and “translation, ribosomal structure and biogenesis” (3,778 unigenes). The categories of “nuclear structure” (31 unigenes) and “extracellular structures” (50 unigenes) had the fewest genes.

The 77,313 annotated sequences were mapped to the reference canonical pathways in the KEGG database. Among these, 56,270 unigenes were assigned to 256 KEGG pathways. The pathways most strongly represented were “metabolic pathways” (6,277 unigenes), “regulation of actin cytoskeleton” (2,722 unigenes), and “pathways in cancer” (2,648 unigenes). Moreover, 78,790 unigene coding sequences (CDSs) were predicted in the transcriptome of scaleless carp, of which 76,208 unigenes were generated through BLAST analysis, and 2,582 unigenes were annotated using the ESTscan program.

### Identification and annotation of potentially differently expressed genes

A total of 8,551 unigenes were identified in the gill libraries of both ecotypes including 4,722 up-regulated and 3,829 down-regulated unigenes (Fig. S1). Among these, 327 unigenes were only expressed in the saltwater ecotype (*G. p. przewalskii*) and 551 unigenes were exclusively detected in the freshwater ecotype (*G. p. ganzihonensis*). In the kidney, 10,910 unigenes were identified in both ecotype libraries, including 4,470 up-regulated and 6,440 down-regulated unigenes (Fig. S2). Among these, 433 unigenes were only observed in the saltwater ecotype, whereas 691 unigenes were only detected in the freshwater ecotype.

An enrichment analysis of the gill was conducted to clarify the biological functions of the differentially-expressed loci. The results indicated that 9,429 loci were enriched in 57 GO terms (*P*-value ≤ 0.05). Among these GO categories, “response to stimulus” (1,207 unigenes), “catalytic activity” (1,110 unigenes) and “immune system process” (289 unigenes) were significantly enriched among the unigenes compared to the whole transcriptome background. In total, 4,026 unigenes were enriched in 248 metabolic pathways (*P*-value ≤ 0.05). The most enriched pathways were “metabolic pathways” (459 unigenes, 11.4%), “focal adhesion” (324 unigenes, 8.05%), and “amoebiasis” (295 unigenes, 7.33%). The same analysis was conducted for the unigenes identified in the kidney. The results indicated that 12,034 unigenes were enriched in 60 GO terms (*P*-value ≤ 0.05). Among these GO categories, “response to stimulus” (4,646 unigenes), “catalytic activity” (4,325 unigenes) and “immune system process” (1,021 unigenes) were significantly enriched among the unigenes compared to the whole transcriptome background. In total, 4,921 unigenes were enriched in 253 pathways (*P*-value ≤ 0.05). The most enriched pathways were “metabolic pathways” (601 unigenes, 12.21%), “focal adhesion” (240 unigenes, 4.9%), and “regulation of actin cytoskeleton” (241 unigenes, 4.9%).

### Identification of putative orthologs between *G. p. przewalskii* and *G. p. ganzihonensi**s***

We recovered 80,916 CDSs for *G. p. przewalskii*, which was slightly higher than the number of CDSs (80,305 CDSs) recovered from *G. p. ganzihonensis*. The average CDS length for *G. p. przewalskii* and *G. p. ganzihonensis* was 613 and 602 bp, respectively. We identified 56,629 pairs of orthologs using a reciprocal best hit BLAST search. A total of 25,631 pairs of orthologous ESTs matched the ORFs of characterized and uncharacterized proteins. The median length of these transcripts was 1,238 bp, ranging from 163 to 16,276 bp.

### *K*_a_/*K*_s_ between pairs of orthologs

The *K*_a_/*K*_s_ ratio is used as an indicator of selection acting on a protein-coding gene^20^. Among the 25,631 pairs of orthologs identified, divergence was sufficiently high for 1,546 orthologs (6.0%), facilitating the calculation of both *K*_a_ and *K*_s_ rates. Among the orthologous pairs for which *K*_a_/*K*_s_ values could be calculated, 242 pairs (15.7%) had a *K*_a_/*K*_s_ > 1, indicating positive selection, and 357 pairs (23.1%) had a *K*_a_/*K*_s_ between 0.5 and 1, indicating weak purifying selection (Fig. 5). The vast majority of EST pairs with *K*_a_/*K*_s_ > 1 were associated with the GO category of “immune response”. For the remaining orthologous ESTs, we could only calculate either *K*_a_ (2,628 orthologous ESTs, 10.3%) or *K*_s_ (6,349 orthologous ESTs, 24.8%), or the orthologous ESTs were identical (8,344, 32.6%).

### Experimental validation

PCR amplification showed that all qPCR primers produced only single fragments of the expected lengths. The qPCR results for 24 selected unigenes were consistent with the transcript abundance determined using RNA-seq, supporting the reliability of the EST data (Figs. S3 and S4).

## Discussion

A central goal of evolutionary genetics is to identify the genes involved in adaptation to new or changing environments, but the difficulty is that adaptation typically involves many (interacting) genes involved in a multitude of biological functions (signaling pathways, cellular and bodily functions, the immune system, etc.). The introduction of NGS techniques has provided a promising tool to assess expression across the genome and address the goal of linking genes with adaptation to particular habitats. In the present study, the transcriptomes of two ecotypes of scaleless carp were sequenced to investigate the adaptations necessary shift from saline to freshwater in these species. Many candidate genes were identified in several of pathways/functions (e.g., calcium signaling pathway(s), aquaporins (AQPs), the neuroendocrine system, immune defense and apoptosis).

Salinity change is a key driving force for the adaptation of fishes^11^,^21^,^22^,^23^, and ion channels and aquaporins are proteins that are thought to play a role as salinity stress effectors^24^. Ion channels mediate the ionic steady state not only for Na^+^, K^+^ and Cl^−^ but also for divalent cations, such as Ca^2+^. Notably, we observed that the expression of Ca^2+^-binding proteins was significantly down-regulated in the lake ecotype. Calcium-binding proteins participate in many cellular regulatory processes, including metabolism, apoptosis, nerve growth, the immune response and other biological processes^25^. In addition to maintaining ionic homeostasis under salt and alkaline stress, the fish from Lake Qinghai showed improved water homeostasis through the use of AQPs. The functions of AQPs have been studied in various animals, plants and bacteria^26^, but little is known about the role of these proteins in adaptation to salinity and alkalinity in fish. We observed the decreased expression of three AQPs in the gill of the lake ecotype (Table 1). In addition, inositol monophosphatase 1 (IMPA1) was under positive selection in the lake ecotype (Table 2),suggesting an important role for this protein after ecological transitions from saline to freshwater habitats. IMPA1 is essential for the synthesis of inositol, a polyol that plays a protective role in salinity adaptation^27^.

The neuroendocrine system also plays a crucial role in adaptation to different salinity environments. Cortisol is a vital hormone for seawater adaptation in teleost species, and prolactin plays an important role in the adaptation of anadromous (e.g., salmon) and catadromous (e.g., eel) fish^28^. A recent study has demonstrated the involvement of the growth hormone (GH)/insulin-like growth factor I (IGFI) axis in salinity adaptation in euryhaline fish^29^,^30^. A previous study in scaleless carp showed that both plasma GH and IGFI levels transiently increased after transfer from river-water to lake-water^31^. In the present study, the expression levels of GH, IGFI and their receptors significantly differed, providing further evidence that these hormones are involved in the regulation of ion homeostasis and salinity acclimation.

The immune functions of fishes are known to correlate with habitat structure^32^,^33^,^34^. After the colonization of novel habitats or upon the exploitation of vacant ecological niches, selection on immune-related genes can be particularly strong when fishes encounter novel parasites^33^. In the present study, the vast majority of genes under balancing or positive selection (*K*_a_/*K*_s_ > 1) were involved in the immune response, including the interferon regulatory factor 5 and complement component C6 genes. Stress can suppress immune system functions^35^,^36^, and these data suggest a dramatic stress-related immune response. For example, the up-regulated genes involved in the immune response (e.g., acute phase response proteins, inflammatory response and chemotaxis) have been associated with signaling pathways in innate immunity (e.g., up-regulation of C-type lectin gene and complement C4) or implicated in apoptosis (e.g., up-regulation of caspase 3; Table 1). In contrast to these up-regulated genes, the genes associated with antimicrobial immunity (e.g., rhamnose-binding lectin and Jun B) and apoptosis induction (e.g., caspases and programmed cell death 1 ligand 1) were down-regulated.

Although most of the genes under positive selection were immune-related, no significant differences were detected in nephrosin expression. However, strong evolutionary forces were acting on the nephrosin precursor (Table 2). Nephrosin, a newly discovered member of the astacin family, is a gene potentially involved in the regulation of the immune system^37^. Nephrosin is primarily synthesized in the lymphohematopoietic tissues of teleosts, which include the head kidney, kidney and spleen^38^. Boutet *et al.*^39^ used suppression subtractive hybridization (SSH) to show that the immune system (nephrosin) in the gill and intestine is regulated through salinity and our results support the importance of nephrosin in habitat shifts between salt and fresh water.

In summary, we compared transcriptome sequence divergence between freshwater and saltwater ecotypes of scaleless carp (*G. przewalskii*) and these results provide insight into local adaptation and potentially incipient ecological speciation. In addition, these results suggest that a few core genes, involved in immune defense, the neuroendocrine system, the calcium signaling pathway, apoptosis, ion exchange and water absorption, play crucial roles in fish habitat shifts from saline to freshwater. Thus, the present study shows the potential use of NGS transcriptomics to identify the underlying genetics of adaptive traits in wild species, a research field of steadily increasing importance.

## Materials and methods

### Fish sampling and RNA extraction

**Figure 1:**
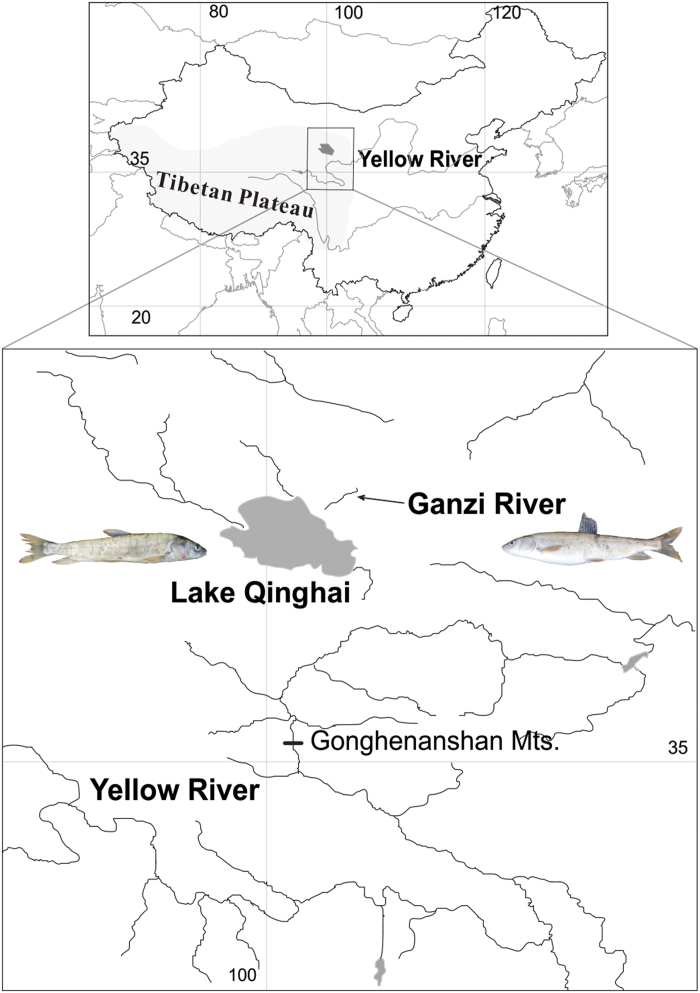
Sampling sites Samples of *Gymnocypris przewalskii przewalskii* were collected from Lake Qinghai (salt water, salinity 14 parts per thousand by weight), and samples of *G. p. ganzihonensis* were collected from the Ganzi River (fresh water). The map was created using ArcGIS version 10.0. Original photographs of fishes by Kai Zhao.

**Figure 2:**
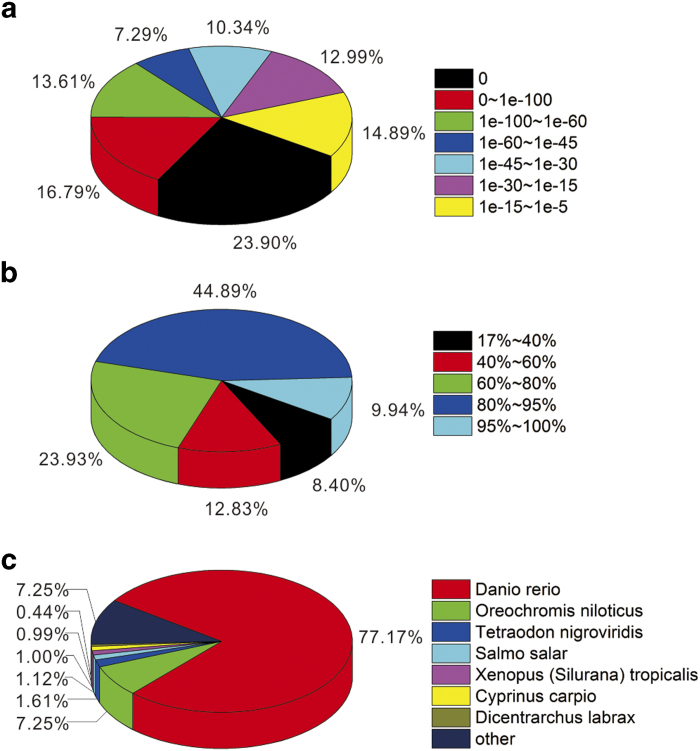
Homology search of assembled sequences against the Nr database. (**a**) E-value distribution of best BLASTX hits for each sequence with a cut-off E-value of 1.0E-5. (**b**) Similarity distribution of the best BLAST hits for each sequence. (**c**) The species distribution is shown as a percentage of the total homologous sequences with an E-value of at least 1.0E-5.

**Figure 3:**
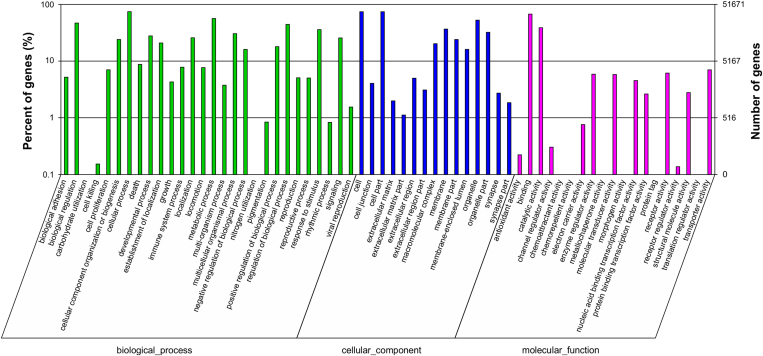
GO putative functions of the transcripts expressed in ***Gymnocypris przewalskii przewalskii*** **and** ***G. p. ganzihonensis***. The transcripts are categorized in three main categories: biological process, cellular component and molecular function. In total, 51,671 unigenes with BLAST matches to known proteins were assigned to a particular gene ontology.

**Figure 4:**
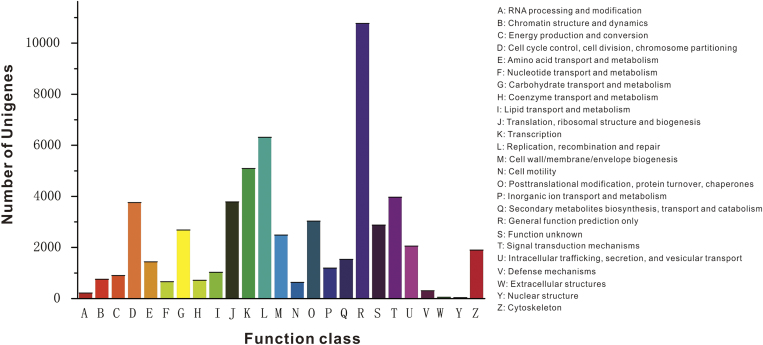
COG classification of the putative proteins. All 25,272 putative proteins with significant similarity to those in the COG database were functionally classified into 25 molecular families. The y-axis indicates the number of unigenes in a specific functional cluster.

**Figure 5:**
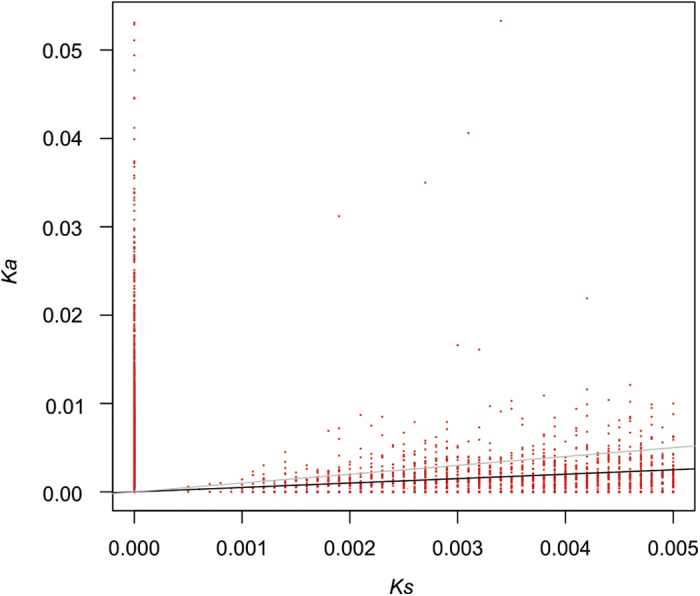
Distribution of *K*_a_ and *K*_s_ Orthologous pairs with *K*_a_/*K*_s_ ratios > 1 are shown above the gray line, whereas pairs with *K*_a_/*K*_s_ ratios between 0.5-1 are shown between the gray and black lines.

Using gill nets, we collected 3 male and 3 female scaleless carp from Lake Qinghai (37°03´N, 100°26´E) and the Ganzi River (37°04´N, 100°27´E) (Fig. 1). The length and weight of the fish were measured (Table S3). Immediately after collection, the fish were dissected, and portions of the gill and kidney were steeped in liquid nitrogen and stored at −80°C. Total RNA was extracted from the frozen gill and kidney tissues using Trizol Reagent (Invitrogen, Carlsbad, CA, USA), according to the manufacturer’s instructions and analyzed for integrity and purity using agarose gel electrophoresis and a NanoDrop 2000 spectrophotometer, respectively.

### Library construction and Illumina sequencing

RNA quality and quantity were verified using an Agilent 2100 Bioanalyzer prior to further processing. Total RNA from each fish was mixed in equal proportions for each ecotype group (n = 6 per group). Subsequently, beads coated with oligo (dT) were used to isolate poly (A) mRNA. Fragmentation buffer (Ambion, Austin, TX, USA) was added to generate short mRNA fragments through digestion. The first strand cDNA was synthesized using random hexamer primers, followed by synthesis of the second strand. The short fragments were purified using the QiaQuick PCR Purification kit (Qiagen, Valencia, CA, USA) for both end repair and poly (A) addition reactions. The purified cDNA libraries were PCR amplified using 18 cycles. The libraries were then sequenced on an Illumina HiSeq™ 2000 platform. The four cDNA libraries were prepared and sequenced independently in the Beijing Genomics Institute in Shenzhen, China.

### *De novo* assembly of mRNA-seq data and functional annotation

The adapter sequences were removed, and reads containing ambiguous bases ‘N’ > 5% or showing low-quality (more than 20% Q ≤ 10 bases) were removed. The *de novo* assembly of the clean reads was performed using the Trinity assembly program^40^. For each library, short reads were first assembled into contigs. Subsequently, the reads with overlapping regions were mapped back to contigs, using the distance of the paired-end reads as a guide. Unknown sequences were represented with ‘N’s, and the unigenes were generated. Subsequently, the unigenes from four libraries (two libraries for each ecotype) were further spliced and assembled to obtain individual non-redundant unigenes through TGICL, with a minimum overlap length of 100 bp^41^.

The unigene functions were annotated through BLAST analysis (with an E-value threshold of 10^−5^) to protein databases, including the NCBI non-redundant (Nr) database, the Swiss-Prot protein database, the Kyoto Encyclopedia of Genes and Genomes (KEGG) database^42^, the Clusters of Orthologous Groups of proteins (COG) database and nucleotide (nt) database. The unigene sequences were also translated into amino acid sequences using ESTScan^43^. Blast2GO^44^ was used to obtain GO annotation of the unigenes based on BLASTX hits against the NCBI Nr database (E-value < 10^−5^). WEGO^45^ was used for GO functional analyses. The unigene sequences were also aligned to the COG database to predict and classify functions. Pathway assignments were generated using the KEGG database and the BLASTX algorithm with an E-value threshold of 10^−5^.

### Identification of differentially expressed genes

The gene expression levels were measured in the RNA-seq analyses as the number of reads per kilobase of the exons in a given gene per million mapped reads (RPKM)^46^. To identify genes and alleles associated with a saline niche, we determined the number of reads for each coding region in the four sample libraries, and subsequently calculated the ratio of reads in the same tissue between the two ecotypes. The statistical significance of the differential expression value for each gene was determined using the methods of Audic and Claverie^47^, and the results of the statistical tests were corrected for multiple testing using the Benjamini–Hochberg false discovery rate (FDR). The expression of a particular sequence was considered significantly different when the adjusted *P*-value obtained using this method was ≤0.001 and there was at least a two-fold change (≥1 or ≤−1 in log2 ratio value) in the sequence count between two libraries. After screening differentially expressed genes (DEGs), GO function and KEGG pathway analyses were performed.

### Identification of orthologous genes

We used the bidirectional best-hit method in BLAST, with a bit score threshold of ≥300, to identify putatively orthologous ESTs between the two ecotypes. This bidirectional best hit threshold requires that the alignment of any two unigenes is longer than 150 bp. The bidirectional best hit method has been widely used to identify putative orthologous genes between closely related species^48^,^49^. Open reading frames (ORF) for the putatively orthologous ESTs were determined through a BLASTX analysis (NCBI blast version, 2.2.19)^50^ against all known vertebrate proteins from the Universal Protein Resource (UniProt Consortium 2008) and protein data sets for six fishes (*Danio rerio*, *Latimeria chalumnae*, *Tetraodon nigroviridis*, *Oryzias latipes*, *Gadus morhuaand* and *Branchiostoma floridae*) in the Ensembl database (Ensembl 52) using an E-value <10^−5^. If both orthologous ESTs could be annotated, then the coding regions were extracted according to the BLASTX results. The coding sequences were aligned using ClustalW 2.0^51^. As *de novo* transcriptome assemblies do not generally distinguish members of gene families, using bidirectional best hits to identify the orthologous genes might also generate large numbers of false positive orthologous pairs^52^. Therefore, these putative orthologous genes were further matched to the ORFs of known or unknown proteins to remove potential paralogs. If two sequences were identified as paralogous in the BLAST search, then these sequences will likely match to different ORFs. Only pairs of sequences unambiguously mapped to the same ORF with an E-value <10^−5^ were selected as orthologous genes.

### Test for positive selection

We estimated the rate of nonsynonymous substitutions per nonsynonymous site (*K*_a_) to the number of synonymous substitutions per synonymous site (*K*_s_) among putatively orthologous coding regions using the maximum-likelihood method^53^ implemented in yn00 in PAML (ver. 4.0)^54^. Orthologous ESTs with a *K*_s_ rate > 0.005 were excluded from further analyses to avoid analyzing potentially paralogous genes^55^. The genes with *K*_a_/*K*_s_ > 1 were subjected to GO functional and KEGG pathway analyses. Statistical significance was determined using the chi square test (Fisher’s exact test was used when the expected value was less than 5), and the results of all statistical analyses were corrected for multiple testing using FDR.

### Real-time quantitative RT–PCR validation

RNA samples from three biological replicates were used for a real-time quantitative reverse transcriptase PCR (qRT-PCR) validation of the transcriptome data. RNAs used in transcriptome sequencing were converted into cDNAs using the PrimeScript™ RT reagent Kit (Takara, Dalian, China) according to the manufacturer’s instructions. qRT-PCR was performed with SYBR green fluorescent dye using an ABI ViiA™7 (Applied Biosystems, Foster City, CA, USA). The fold-induction values were calculated using the 2^−ΔΔCt^ method according to the manufacturer’s instructions. The primers used in the qRT-PCR analysis are listed in Table S4, and β-actin served as the internal control for the expression analyses.

### Ethics statements

All necessary permits for collection and experimentation were acquired for the described field study from the Agriculture Department of Qinghai Province, China. All samples of fish used in this study followed the guidelines of the regulations of experiments on animals, and was approved by China Zoological Society.

## Author Contributions

R.Z., Z.P. and K.Z. designed the experiment; R.Z., G.L., Y.T. and K.Z. performed the experiment; R.Z., A.L., C.Z., C.T. and Z.P. analysed and interpreted the data; and R.Z., A.L., Z.P. and K.Z. wrote the paper. All co-authors participated in the scientific discussions and commented on the manuscript.

## Additional Information

**Accession codes**: Raw sequencing reads are available at NCBI SRA under accession numbers: SRX673786, SRX673788, SRX673790 and SRX673793.

**How to cite this article**: Zhang, R. *et al.* Local adaptation of Gymnocypris przewalskii (Cyprinidae) on the Tibetan Plateau. *Sci. Rep.* **5**, 09780; doi: 10.1038/srep09780 (2015).

## Supplementary Material

Supplementary Information

## Figures and Tables

**Table 1:** Aquaporins and immune-related gene expression in salt and fresh water ecotypes of *G. przewalskii*. PG = the gill of *G. p. przewalskii*. GG = the gill of * G. p. ganzihonensis*. PK = kidney of *G. p. przewalskii*. GK = kidney of * G. p. ganzihonensis*.

		**mRNA expression level (RPKM)**			
**Name**	**Gene ID**	**PG**	**GG**	**PK**	**GK**
Aquaporin 1a, tandem duplicate 2	Unigene1210_All	1.3880	6.2828	0.7962	7.9713
Aquaporin 8a	Unigene71646_All	1.4999	6.7802		
Aquaporin 8a	Unigene55974_All	1.9734	12.0018		
Aquaporin 1a, tandem duplicate 1	Unigene15184_All			175.6014	17.8940
Aquaporin 3	Unigene38172_All			0.3695	2.6133
Aquaporin 9	Unigene53376_All			0.5674	4.4428
Caspase 3	CL19429.Contig2_All	1.5626	3.2189	1.6890	3.4840
Complement C4-1	Unigene36760_All	19.0162	51.3929		
Complement C4-2	Unigene8552_All	1.0666	3.1018		
C-type lectin	Unigene49896_All	1503.6586	3058.8138	2.1484	5.8732
Jun B	Unigene51386_All	257.8937	90.3734	190.7652	88.2530
Programmed cell death 1 ligand 1	CL8566.Contig2_All	6.0275	2.3540	4.4182	1.4804
Rhamnose binding lectin	Unigene29785_All	4.1940	0.1490		

**Table 2:** List of selected candidate genes under positive selection.

Protein homolog	*K*_a_/*K*_s_ ratio
Nephrosin precursor	5.8196
Inositol(myo)-1(or 4)-monophosphatase 1	3.8012
Potassium voltage-gated channel subfamily C member 3-like	3.6840
Immediate early response 5	2.8468
NAD(P)H dehydrogenase, quinone 1	1.4015
Solute carrier family 22, member 5	1.2610
Glutamine synthetase	1.2067
Glutathione synthetase	1.1145
Complement component C6	1.0280
Interferon regulatory factor 5	1.0062
