# Supplementary material for: Local adaptation of Gymnocypris przewalskii (Cyprinidae) on the Tibetan Plateau
Source: Sci Rep. 2015 May 6;5:9780. doi: 10.1038/srep09780 (PMC4421831; doi:10.1038/srep09780)
Supplement: Supplementary Information [file srep09780-s1.doc]

**Supplementary information**

**Local adaptation of** ***Gymnocypris przewalskii* (Cyprinidae)** **on the Tibetan Plateau**

Renyi Zhang^1,4^, Arne Ludwig^2^, Cunfang Zhang^1^, Chao Tong^1,4^, Guogang Li^1,4^, Yongtao Tang^1,4^, Zuogang Peng^3,*^ & Kai Zhao^1,*^

^1^Key Laboratory of Adaptation and Evolution of Plateau Biota, Northwest Institute of Plateau Biology, Chinese Academy of Sciences, Xining 810001, China;

^2^Department of Evolutionary Genetics, Leibniz Institute for Zoo and Wildlife Research, Berlin 10324, Germany;

^3^Key Laboratory of Freshwater Fish Reproduction and Development (Ministry of Education), Southwest University School of Life Sciences, Chongqing 400715, China;

^4^University of Chinese Academy of Sciences, Beijing 100049, China


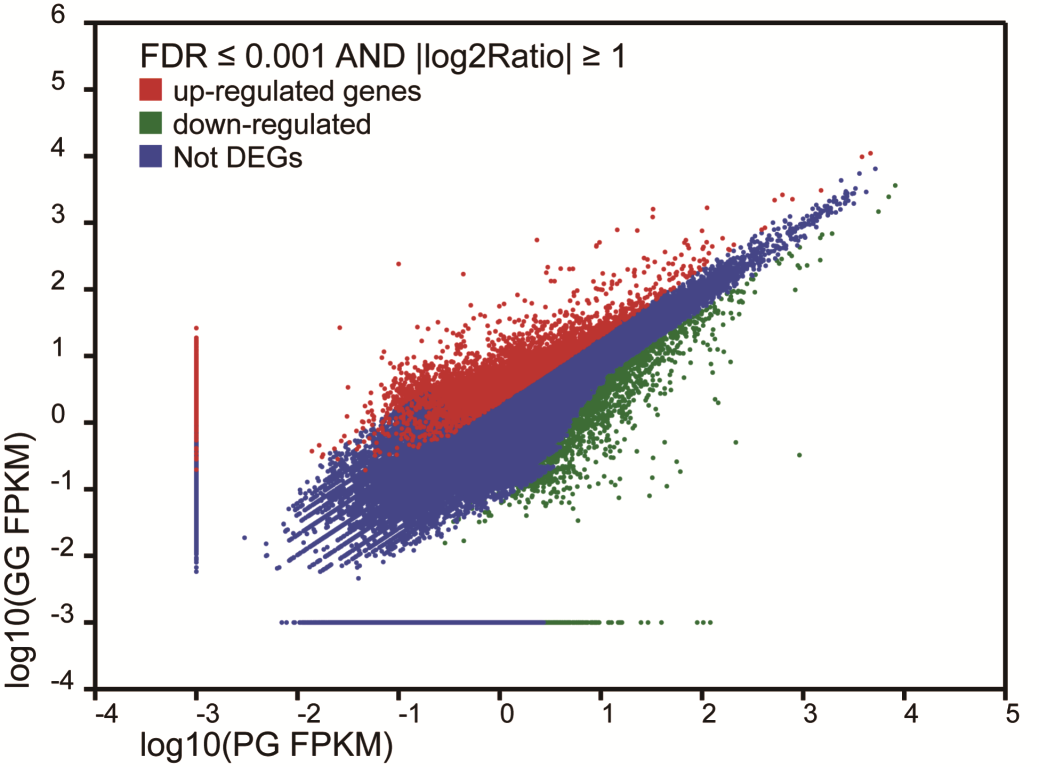


**Fig. S1: Differentially expressed genes (DEGs) between *Gymnocypris przewalskii przewalskii* and *G. p. ganzihonensis* in gill tissue.** DEGs were determined using a threshold of FDR ≤ 0.001 and |log2Ratio| ≥ 1. The red spots represent up-regulated DEGs, and the green spots represent down-regulated DEGs. The blue spots indicate unigenes that did not show obvious changes between *G. p. przewalskii* and *G. p. przewalskii*. PG: the gill of *G. p. przewalskii*. GG: the gill of *G. p. przewalskii*.

**
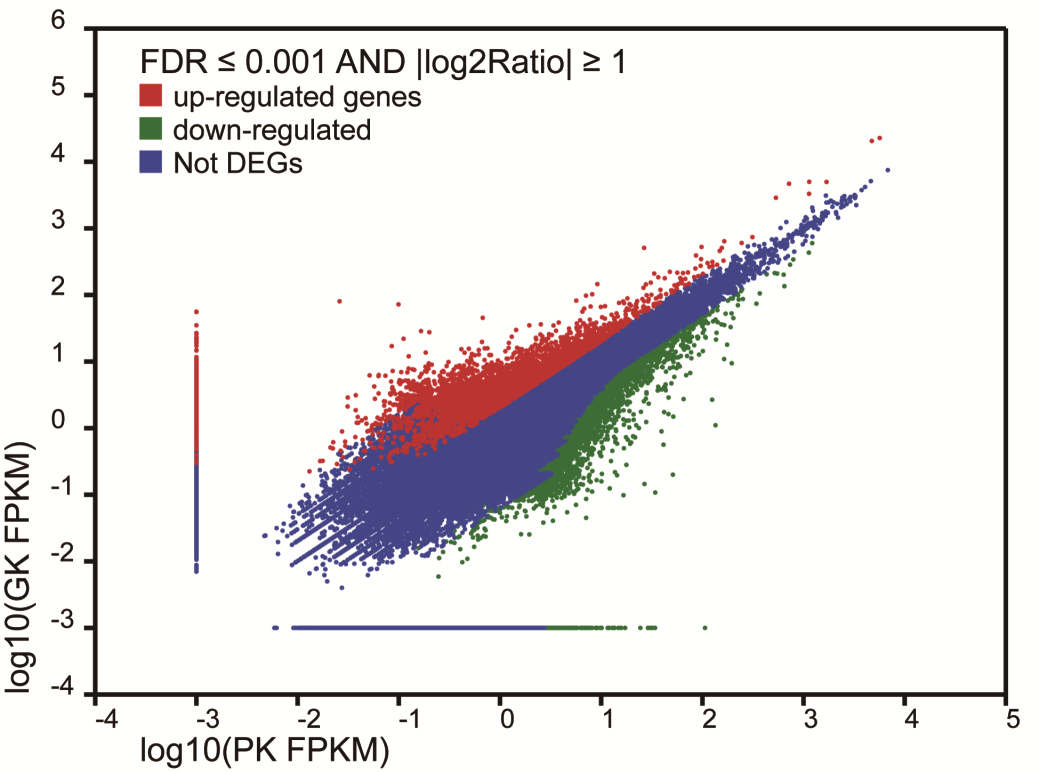
**

**Fig. S2: DEGs between *Gymnocypris przewalskii przewalskii* and *G. p. ganzihonensis* in kidney tissue.** DEGs were determined using a threshold of FDR ≤ 0.001 and |log2Ratio| ≥ 1. The red spots represent up-regulated DEGs, and the green spots represent down-regulated DEGs. The blue spots indicate unigenes that did not show obvious changes between *G. p. przewalskii* and *G. p. przewalskii*. PK: kidney of *G. p. przewalskii*. GK: kidney of *G. p. przewalskii*.

**
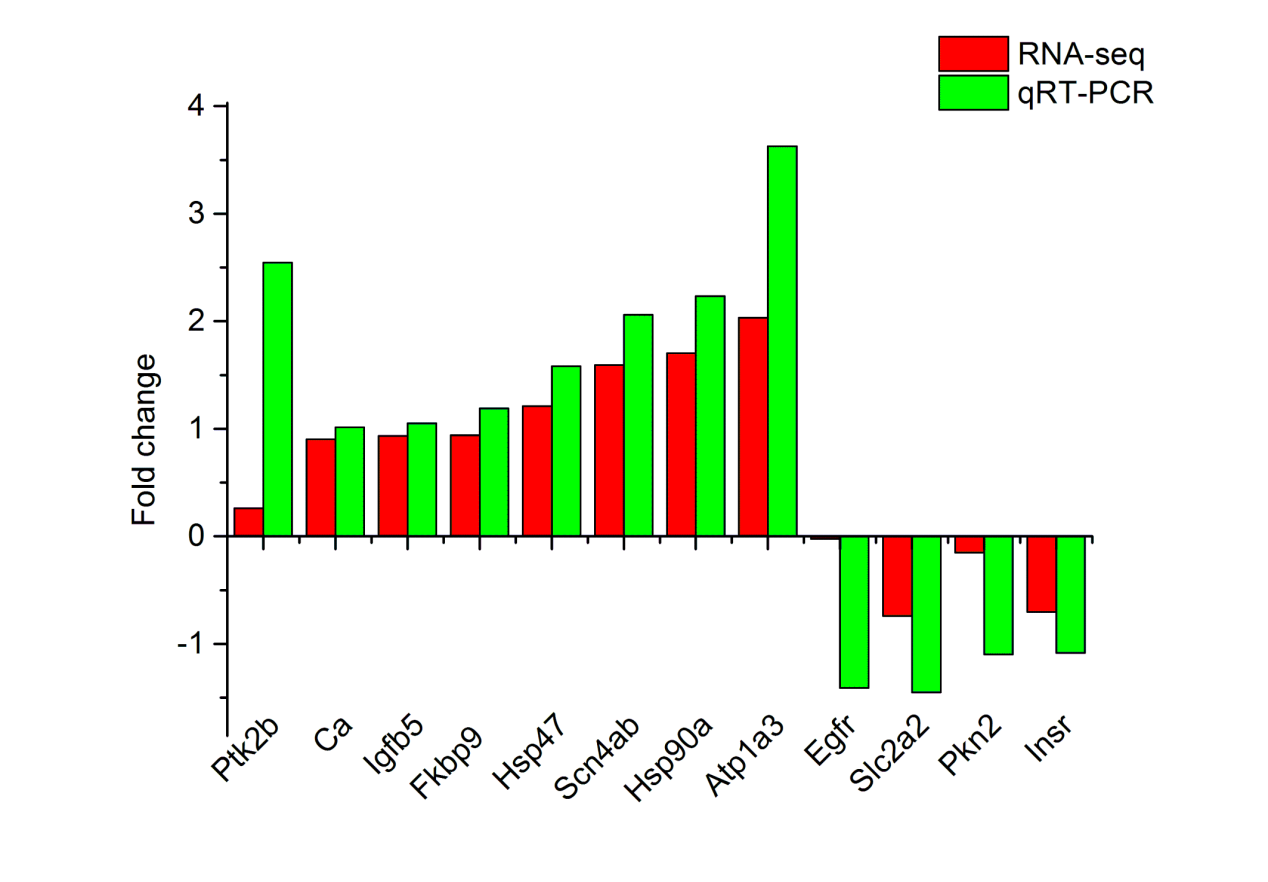
**

**Fig. S3: qRT-PCR validation of the expression of selected genes in gill tissue.** Changes in the transcript levels of 12 selected genes were detected using qRT-PCR. The x-axis shows the fold-change in the transcript abundance of the unigenes. The red bar indicates transcript abundance changes calculated using the RPKM method. The green bar represents relative expression levels determined through qRT-PCR using the 2^-ΔΔCT^ method. Ptk2b: Protein-tyrosine kinase 2-beta. Ca: Carbonic anhydrase. Igfb5: Insulin-like growth factor binding protein 5. Fkbp9: FK506 binding protein 9. Hsp47: Heat shock protein 47. Scn4ab: Sodium channel, voltage-gated, type IV, alpha, b, Hsp90a: Heat shock protein 90 alpha. Atp1a3: ATPase, Na^+^/K^+^ transporting, alpha 3 polypeptide. Egfr: Epidermal growth factor receptor. Slc2a2: Solute carrier family 9 (sodium/hydrogen exchanger), member 2. Pkn: Serine/threonine-protein kinase N2. Insr: Insulin receptor.


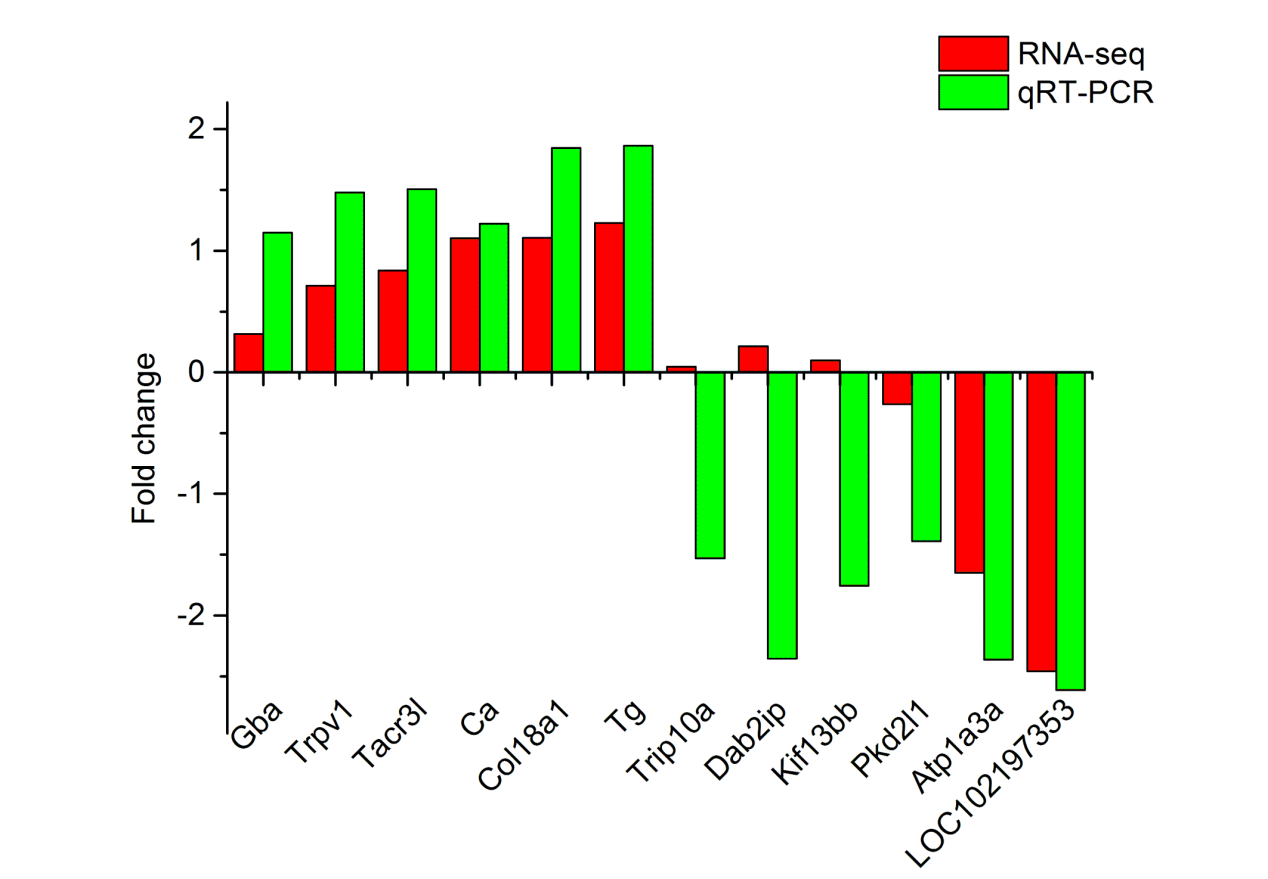


**Fig. S4: qRT-PCR validation of the expression of selected genes in kidney tissue.** Changes in the transcript levels of 12 selected genes were detected using qRT-PCR. The x-axis shows fold-change in the transcript abundance of the unigenes. The red bar indicates transcript abundance changes calculated using the RPKM method. The green bar represents the relative expression level determined through qRT-PCR using the 2^-ΔΔCT^ method. Gba: Glucosidase, beta, acid. Trpv1: Transient receptor potential cation channel, subfamily V, member 1. Tacr3l: Tachykinin receptor 3-like. Ca: Carbonic anhydrase. Col18a1: Collagen, type XVIII, alpha 1. Tg: Thyroglobulin. Trip10a: Thyroid hormone receptor interactor 10a. Dab2ip: DAB2 interacting protein. Kif13bb: Kinesin family member 13Bb. Pkd2l1: Polycystic kidney disease 2-like 1. Atp1a3a: ATPase, Na^+^/K^+^ transporting, alpha 3a polypeptide. LOC102197353: Pituitary adenylate cyclase-activating polypeptide type I receptor-like.

**Table S1** Overview of the *de novo* assembly of the *G. przewalskii* transcriptome.

|  | *G. p. przewalskii* | | |  | *G. p. ganzihonensis* | | |
| --- | --- | --- | --- | --- | --- | --- | --- |
|  | Gill (PG) | | Kidney (PK) |  | Gill (GG) | | Kidney (GK) |
| Total clean reads | 82,421,158 | | 81,884,878 |  | 78,605,558 | | 80,382,460 |
| Total clean nucleotides (nt) | 7,417,904,220 | | 7,369,639,020 |  | 7,074,500,220 | | 7,234,421,400 |
| Number of contigs | 243,557 | | 256,575 |  | 239,166 | | 240,762 |
| Mean length(nt) | 314 | | 304 |  | 316 | | 307 |
| N50 length | 547 | | 519 |  | 560 | | 514 |
| Number of unigenes | 135,634 | | 134,156 |  | 132,554 | | 130,604 |
|  | 133,007 | | |  | 129,336 | | |
| Mean length of unigenes (nt) | 748 | 745 | |  | 736 | 704 | |
| N50 length | 1557 | 1532 | |  | 1490 | 1399 | |

**Table S2** Annotation of assembled *G. przewalskii* unigenes.

| Category | Account | Percentage (%)^c^ |
| --- | --- | --- |
| Nr^a^ annotated unigenes | 77,313 | 48.9% |
| nt^b^ database | 115,934 | 73.3% |
| Swissprot | 66,677 | 42.2% |
| GO classified unigenes | 51,671 | 32.7% |
| COG classified unigenes | 25,272 | 16.0% |
| KEGG classified unigenes | 56,270 | 35.6% |
| All | 122,982 | 77.8% |

^a^Nr: NCBI non-redundant sequence database.

^b^nt: NCBI nucleotide sequence database.

^c^Percentage of annotated unigenes in total 158,087 assembled unigenes of coconut.

**Table S3** Information of samples.

| Ecotype | Code | Gender | Weight (g) | Length (cm) | Collection time |
| --- | --- | --- | --- | --- | --- |
| *G. p. przewalskii* | GPP1 | male | 232 | 29 | February 20, 2012 |
| *G. p. przewalskii* | GPP2 | male | 246 | 30 | February 20, 2012 |
| *G. p. przewalskii* | GPP3 | male | 228 | 27 | February 20, 2012 |
| *G. p. przewalskii* | GPP4 | female | 235 | 29 | February 20, 2012 |
| *G. p. przewalskii* | GPP5 | female | 258 | 32 | February 20, 2012 |
| *G. p. przewalskii* | GPP6 | female | 249 | 30 | February 20, 2012 |
| *G. p. ganzihonensis* | GPG1 | male | 232 | 28 | February 20, 2012 |
| *G. p. ganzihonensis* | GPG2 | male | 233 | 27 | February 20, 2012 |
| *G. p. ganzihonensis* | GPG3 | male | 251 | 29 | February 20, 2012 |
| *G. p. ganzihonensis* | GPG4 | female | 261 | 32 | February 20, 2012 |
| *G. p. ganzihonensis* | GPG5 | female | 252 | 30 | February 20, 2012 |
| *G. p. ganzihonensis* | GPG6 | female | 248 | 27 | February 20, 2012 |

**Table S4** Primers used for the qRT-PCR analysis of genes in the *G. przewalskii* gill and kidney transcriptomes.

| Gene ID | Description | Forward primer (5′–3′) | Reverse primer (5′–3′) | Tissue |
| --- | --- | --- | --- | --- |
| Ptk2b | Protein-tyrosine kinase 2-beta | GTGATGGAGATGGTGAAGGTC | GTAGAGTCGGGAGGATGTCGT | gill |
| Ca | Carbonic anhydrase | CAAATCAAAGGGCAGACAGACT | AGAAGAGGAGGTGTGGTCAGAG | gill |
| Igfbp5 | Insulin-like growth factor binding protein 5 | CTAAATGCGTGGCAAGGTAAG | CAGGGCGAAATCAGTAATCAG | gill |
| Fkbp9 | FK506 binding protein 9 | TCAACCACAAGACACAACCAA | AAACTCAAGGCAGATGAAGCA | gill |
| Hsp47 | Heat shock protein 47 | ATGAAGGATTTGGTGTCAGGAG | CACAGTAGCCAGTAAGCACAGG | gill |
| Scn4ab | Sodium channel, voltage-gated, type IV, alpha, b | CCTTTGCTATCGGACATTTGA | TGCCATTATCCTCGTCATCAT | gill |
| Hsp90a | Heat shock protein 90 alpha | TACATCTGGGAGTCTGCTGCT | ACGAAGAGCGTAATGGGGTAG | gill |
| Atp1a3 | ATPase, Na+/K+ transporting, alpha 3 polypeptide | CGCAGAATAAATCCAGCAGAG | ACACAGGGTCTTGCCTCAGTA | gill |
| Egfr | Epidermal growth factor receptor | GAGACTTGGCTGCTCGTAATG | CCATCTGCGTGATACTCCTTC | gill |
| Slc2a2 | Solute carrier family 9 (sodium/hydrogen exchanger), member 2 | CCCTCACGGTCTTCATCACTAT | GTAAATCAGGCATCCACAGAGA | gill |
| Pkn2 | Serine/threonine-protein kinase N2 | GGAGCGGATTGCTGTATTATTC | GCAGGGTAACTGGTCAAAGAGT | gill |
| Insr | Insulin receptor | TCTTATTGGCTTCTGCTTCCTC | GACCTCGTCTCGTCTTTTCACT | gill |
| Gba | Glucosidase, beta, acid | GCTTGAGAAGCTGTTTTGGTG | GGCTGTGAGAAGTTGCGTTTA | kidney |
| Trpv1 | Transient receptor potential cation channel, subfamily V, member 1 | GGAATCTCACTGCCATACACG | GAAGAACTCACCCCACTCACA | kidney |
| Tacr3l | Tachykinin receptor 3-like | TTCAGGTGGTGTCCCTTCAT | GAGTTTAGTCGCTGCTGTGCT | kidney |
| Ca | Carbonic anhydrase | TCTGCCCTTTGATTTGATGTC | TTGCTGTGGTTGGAGTTTTTC | kidney |
| Col18a1 | Collagen, type XVIII, alpha 1 | CCTTCCTGTCCTCCAAACTTC | CATCTCTGCCATCAAACGAGT | kidney |
| Tg | Thyroglobulin | GCTCTTGGGTTGGACTTTGA | TCCTCAGTGTTCCTTCTGGTC | kidney |
| Trip10a | Thyroid hormone receptor interactor 10a | TGAAGTTGTGAAGCGTAGTCG | AGGGAGTGGAGAGAAGCAGAG | kidney |
| Dab2ip | DAB2 interacting protein | GCAAGAGCCTGTCAATGGTAG | GCACCCTCACCTAAACCTTCT | kidney |
| Kif13bb | Kinesin family member 13Bb | TACAGCAGACAGGACAGGAAGA | TACCAGGAGTGGAAGGACTACG | kidney |
| Pkd2l1 | Polycystic kidney disease 2-like 1 | GGAAAACGATGTAGACGAGCA | ATCTGCCTAACCCCTATCTGG | kidney |
| Atp1a3a | ATPase, Na^+^/K^+^ transporting, alpha 3a polypeptide | TCAGCGGGTATCAAAGTCATC | ATCATCCATCTGCTCCTGTGT | Kidney |
| LOC102197353 | Pituitary adenylate cyclase-activating polypeptide type I receptor-like | AGGATGAAGGAGGCAAAGAGA | CGTGGCTGGACAAAAATCTAC | kidney |
| Act | actin, beta, a positive control | GCCAACAGGGAAAAGATGAC | TTGCCAATGGTGATGACCTG | both |
